# Effects of Concentration, Salinity, and Temperature on the Rheological Behavior of Diutan Gum in Aqueous Solution: Analysis and Eventual Applicability for Deep Drilling

**DOI:** 10.1021/acsomega.5c03075

**Published:** 2025-08-07

**Authors:** Valéria S. dos Santos, Andreas Nascimento, Saeed Z. Zadeh, Edson José Soares, Cleocir José Dalmaschio

**Affiliations:** † Programa de Pós-Graduação em Engenharia Mecânica, 28094 Universidade Federal de Itajubá, Itajubá 37500-903, Brazil; ‡ Institute of Drilling Engineering and Fluid Mining (IBF), 26545 TU Bergakademie Freiberg (TUBAF), 09599 Freiberg, Germany; § Labreo - Department of Mechanical Engineering, 28126 Federal University of Espírito Santo (UFES), Vitória 29075-910, Brazil; ∥ Labpol/LabPetro - Department of Chemistry, 28126 Federal University of Espírito Santo (UFES), Vitória 29075-910, Brazil

## Abstract

Diutan gum (DG) is a microbial polysaccharide with great potential for application in activities associated with the deep drilling industry. This study aims to investigate the rheological behavior of diutan gum in aqueous solutions, focusing on its resistance to thermal and saline stresses for potential application in water-based drilling fluids. Thermogravimetric analysis (TGA) was performed to evaluate the thermal stability of the polymer. Two thermal events were observed from TGA: water desorption (approximately 100 °C) and polymer decomposition (230–274 °C, 40% mass loss). The high decomposition onset temperature (230 °C) exceeds typical drilling fluid requirements (<200 °C), confirming excellent thermal stability for high-temperature, high-pressure (HTHP) applications. The rheological behavior in aqueous solutions at different concentrations (0.75–1.75 g/L), salinities (3–90 g/L NaCl), and temperatures (25–80 °C) was studied and analyzed. Accelerated aging, static aging (SA), and dynamic aging (DA) tests (API RP 13B-1) on diutan gum solutions (1.75 g/L) were performed to evaluate salt tolerance (≤180,000 mg/L NaCl), thermal resilience (≤140 °C), and shear resistance (600 rpm) over 16 h, simulating field conditions. Pseudoplastic rheological behavior was observed for most samples, except for the sample subjected to DA at 140 °C and 600 rpm, which exhibited Newtonian behavior. Additionally, the elastic modulus (*G*′ > *G*″) was more significant at low frequencies (0.1–10 Hz), indicating the potential ability of diutan to form more organized lattice structures, as observed under nearly all studied conditions. Rheological results from SA/DA tests confirmed Pseudoplastic behavior under all conditions, demonstrating the stability of diutan gum despite environmental variations. However, its thermomechanical resistance is limited under extreme conditions (140 °C, 600 rpm). The FTIR spectra confirmed the maintenance of the chemical structure of the diutan, which did not suffer from the loss of functional groups. This stability at high salinity and temperature can be attributed to the ability of diutan to form a double helix structure in aqueous media. This conformation drapes water in its interior and protects the main chain and its functional groups from the degradation imposed on the system. Based on its remarkable thermal and saline stability, diutan gum has emerged as a highly effective additive for water-based drilling fluids, improving wellbore stability, cutting suspension, and overall rheological performance under demanding conditions, including deepwater and presalt oil reservoirs.

## Graphical abstract

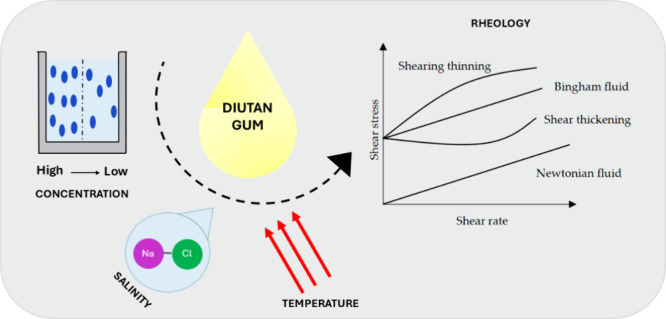

## 1 Introduction

Biopolymers are macromolecules derived from renewable resources in a raw or chemically modified manner.^1,2^ Natural polymers have emerged as substitutes for synthetic polymers, the best known being starch and cellulose from different biodegradable sources, which are low-cost and easy to obtain.^3,4^ A prominent group of polysaccharides **is** those of microbial origin, as they exhibit physicochemical characteristics of interest in thickening, emulsification, stabilization, and gelatinization.^5,6^ Polysaccharides can adopt strong molecular conformations in aqueous solutions that affect their properties and functions.^6^ One of them is the helix-like conformation, which tends to interact with other materials, forming structures that often result in the formation of gels.^7^ Many internal factors influence the conformation adopted, such as the type of monosaccharide, glycosidic bonds, and side chains.^8^ The helices can be single, double, or triple.^9^

Diutan gum is a microbial polysaccharide whose structure is composed of repeated configurations of rhamnose, glucose, and glucuronic acid (β-1,4-L-rhamnopyranosyl, 2-saccharides L-rhamnopyranosyl, β-1,4-d-glucopyranosyl, and β-1,4-3-glucopyranosyl), belonging to the group of polymers with high molecular weights (5.2 × 10^6^ g/mol).^10^ Diutan gum in aqueous solution has a high water-holding capacity, salinity tolerance, suspending power, and thermal stability in the 5–150 °C.^11,12^ Its main feature is the formation of a perfect double helix structure in aqueous media.^11^ In terms of toxicity, studies have shown that diutan gum does not have significant harmful effects on marine ecosystems, including the fauna and flora of marine organisms, including rainbow trout (*Oncorhynchus mykiss*), water fleas (*Daphnia magna*), the copepod *Acartia tonsa*, and the diatoms *Skeletonema costatum* and *Phaeodactylum tricornutum*, which were subjected to prolonged contamination tests, with no observed mortality in any of the test species. Furthermore, no relevant toxicological risks have been observed in its handling by humans, indicating a favorable environmental and occupational safety profile.^13,14^

The carboxylic acid present in the glucuronic acid unit acts as a supplier of anionic charges to the polymer chain.^15^ On the side, two rhamnose units are connected to the second glucose unit, which in aqueous solution forms hydrogen bonds with the carboxylic groups present along the main chain, generating a rigid double helix.^16^ This helical structure in aqueous solution has a conformation similar to that of xanthan gum; however, the main difference between the conformations is that diutan gum is more stable.^17^ This perfect structure endows polysaccharides with good resistance to saline environments and high temperatures.^3^

Regarding industrial applications, its use is more common in the cement industry, where it acts as a thickening agent that effectively maintains free water in cement-based materials and prevents concrete separation.^18^ It has also been reported that diutan gum can be used as a polymeric additive in drilling and cementing fluids, as well as an injection fluid for oil recovery purposes.^19,20^ The complexity of drilled fields has increased, increasing the need for the industry to seek solutions that focus on improving the thermal resistance and rheology of these fluids.^21^ Although the relationship between chemical structure and rheological properties has been explored in different environments for most polysaccharides, studies on the properties of solutions containing diutan gum are rare.^22^ Therefore, an understanding of the behavior of diutan gum in aqueous solutions is essential to improve its use in several industrial processes, especially in its application as a polymeric additive in aqueous drilling fluids for wells with high temperature and salinity, such as the Brazilian presalt layer and some geothermal systems.

presalt rocks can be defined as reservoirs located under an extensive layer of evaporites (known as saline rocks), with temperatures ranging from 55 to 140 °C.^23^ One of the current challenges has been to find aqueous fluids that are more resistant to these severe conditions of thermal degradation and salinity while meeting the growing concern about environmental protection.^15,24,25^ Eco-friendly and chemically stable biopolymers have received considerable attention because they are applied as polymeric additives in aqueous drilling fluids.^26^ Polymers in this type of composition have become indispensable because they aim to improve the fluid’s rheology and filtration characteristics when subjected to high-temperature conditions.^27^

Reference 28 investigated the isolated effects of salinity (140,000–180,000 mg/L NaCl) and temperature (60–140 °C) on the rheological behavior of diutan gum and compared static aging (SA) and dynamic aging (DA, 600 rpm). The results showed that under static conditions, the rheological properties (apparent viscosity and viscoelastic modulus) of the system were maintained even at extreme salinities (180,000 mg/L) or elevated temperatures (140 °C). However, during DA, significant degradation was observed only at 140 °C. The authors did not investigate the molecular mechanisms of this degradation, particularly the combined effect of mechanical shear and chemical reactions on the polymer chain, which could be elucidated through techniques such as FTIR spectroscopy to analyze changes in functional groups.

This study investigated the rheological behavior of diutan gum in aqueous solutions to evaluate its potential as a polymeric additive in aqueous drilling fluids, considering the challenges posed by temperature and salinity, which accelerate the degradation of polysaccharides. Thermogravimetric analysis (TGA) was performed to evaluate the thermal stability of the polymer. Two thermal events were observed from TGA:water desorption (approximately 100 °C) and polymer decomposition (230–274 °C, 40% mass loss). The rheological response was analyzed at different concentrations (0.25–1.75 g/L), salinities (3–90 g/L NaCl), temperatures (25–80 °C), and aging conditions (static and dynamic) via *flow curves* and *small amplitude oscillatory shear* (SAOS). The results showed that diutan gum exhibited pseudoplastic behavior under all of the conditions tested, with a reduction in viscosity (η) as the shear rate increased. In addition, SAOS tests revealed a storage modulus (*G*′) greater than the loss modulus (*G*″) in the frequency range of 0.1–10 Hz, suggesting the formation of a well-organized structural network. Fourier transform infrared (FTIR) spectroscopy confirmed the structural stability of diutan gum under various salinity and temperature conditions, with no evidence of functional group degradation. As a novel contribution beyond the prior work of Santos et al.,^28^ we specifically investigated the effects of DA at 140 °C via FTIR. The results revealed significant spectral changes indicative of polymer backbone hydrolysis and oxidation, leading to a reduced molar mass. This degradation was corroborated by the rheological transition from pseudoplastic to Newtonian behavior, demonstrating the critical impact of combined mechanical shear and high temperature on polymer stability.

## 2 Experimental Sections

### 2.1 Reagents

The reagents used in the experiments, diutan gum (average molecular weight of 5.2 × 10^6^ g/mol and intrinsic viscosity of 5450 mL g^–1^ at 25 °C) and sodium chloride (NaCl), were supplied by the companies CP Kelco (United States) and Dinamica (Brazil), respectively. All products were used without further purification.

### 2.2 Thermogravimetric Analysis (TGA)

TGA was performed by using a Shimadzu DTG-60 thermal analyzer under a dynamic nitrogen atmosphere (50 mL/min flow rate). Samples weighing approximately 6 mg were heated from ambient temperature to 900 °C in a platinum crucible.

### 2.3 Preparation of Aqueous Solutions

All aqueous solutions were prepared from a basic step: dry diutan gum was added to distilled water, and the mixture was stirred at 15,000 rpm for 10 min in an Ultra Turrax (model IKA T25). The samples were subsequently allowed to rest for 24 h to cure their rheological properties.^29^ Solutions with various polymer concentrations were prepared at 0.25, 0.75, 1.00, 1.25, 1.50, and 1.75 g/L at 25 °C. The samples for the temperature and salinity tests were prepared at a fixed concentration of 1.75 g/L at 25 °C.^4,11^

### 2.4 Rheology

The rheological characterization was divided into flow curves and SAOS tests. In step 1, rheological measurements were performed in a Haake Mars III rotational rheometer (Thermo Scientific, Germany) equipped with a concentric cylinder geometry (CC25/Ti; inner diameter = 25 mm, gap = 1 mm) operating in controlled stress (CS) mode. Measurements were performed under thermostatic control (±0.1 °C) using a thermal bath, and data acquisition was carried out via RheoWin software.^30^ Oscillatory tests were performed using the same system but with a plate–plate geometry (PP60/Ti; diameter = 60 mm, gap = 0.5 mm) operating in controlled stress and strain modes. All measurements were conducted at 25 ± 0.1 °C, with a fixed oscillatory stress of 0.5 Pa (previously determined via amplitude sweep to ensure testing within the linear viscoelastic region).^31^ The tests for viscosity and SAOS for analyzing the effects of temperature were performed at 25, 40, 60, and 80 °C. Peltier performed all of the thermal control.

### 2.5 Static and Dynamic Aging

Aging tests were conducted in a sealed metallic reactor under controlled temperature (60 and 140 °C) and salinity (140,000 and 180,000 mg/L NaCl) conditions. SA was performed without agitation, whereas DA employed continuous mixing at 600 rpm. All tests used a 120 mL sample volume and lasted 16 h, following API RP 13B-1 guidelines. Subsequent rheological characterization (viscosity and viscoelasticity) was performed as described in Section 2.4 (Rheology) at 25 °C.

### 2.6 FTIR

Samples of diutan gum aqueous solutions at a concentration of 1.75 g/L subjected to different temperatures (25, 40, 60, and 80 °C), salinities (3, 30, 60, and 90 g/L), and aging (static at 140 °C and dynamic at 140 °C and 600 rpm) were dried under vacuum in an oven. The oven was connected to a vacuum pump for 24 h to remove the water present in the aqueous solution, enabling the formation of a dry solid. For the solid samples, FTIR spectra were obtained on an Agilent Cary 630 FTIR spectrometer with ZnSe crystals at a resolution of 4 cm^–1^ and an accumulation of 128 scans in ATR mode.

## 3 Results and Discussion

### 3.1 Thermogravimetric Analysis

Figure 1 shows the TGA results for the diutan gum. TGA was performed after the samples were heated from ambient temperature to 900 °C.

**Figure 1:**
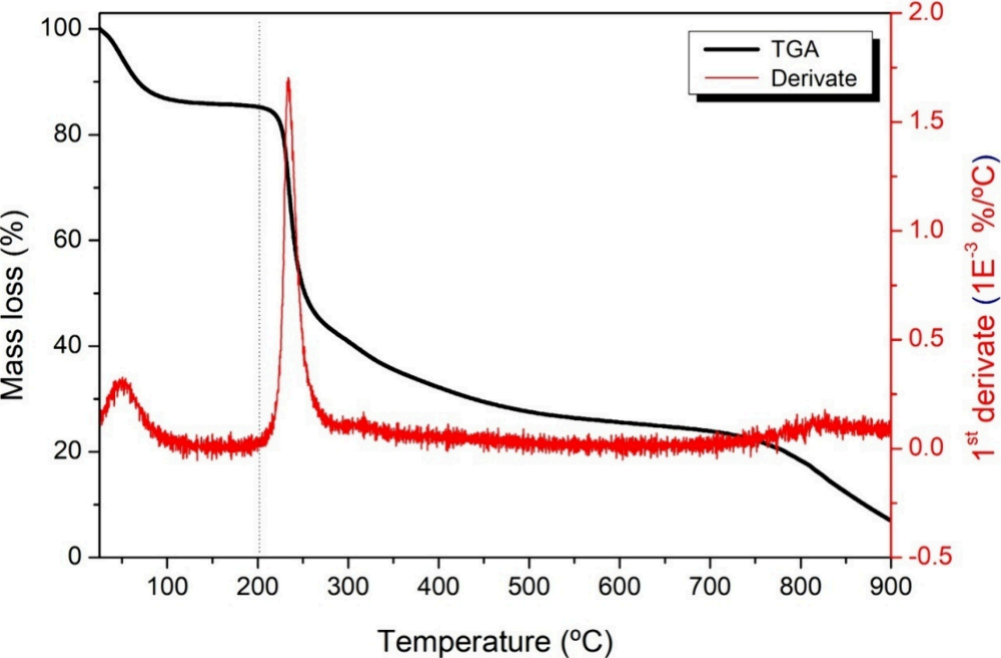
Thermogravimetric analysis (TGA) of diutan gum heated from ambient temperature to 900 °C in a platinum crucible (TGA curve obtained under a nitrogen atmosphere with a flow rate of 50 mL·min^–1^ and a heating rate of 10 °C·min^–1^; the sample mass was approximately 6 mg).

TGA revealed two distinct stages of mass loss in diutan gum. The first stage, observed from room temperature until approximately 100 °C, corresponds to the physical desorption of water adsorbed on the material’s surface, a typical characteristic of hydrophilic polysaccharides. The second stage, beginning at 230 °C, with a maximum peak in the DTG at 274 °C, shows a significant mass loss of approximately 40%, which is associated with the thermal decomposition of the main polymeric structure. The significant decomposition temperature (230 °C) exceeds the conventional operational range of drilling fluids (typically <200 °C), indicating its suitability for deep-well and high-temperature operations.

### 3.2 Effect of Concentration

Figure 2 shows the relationship between the apparent viscosity (η) and shear rate (γ̇) for aqueous solutions of diutan gum with concentrations ranging from 0.25 to 1.75 g/L (a concentration of 0.25 g/L was established as a baseline to systematically evaluate how increasing concentrations affect the viscous behavior of diutan gum in aqueous solution). The solutions exhibited a power-law region for all concentrations, in which the apparent viscosity gradually decreased with increasing shear rate.^18^ Fluids with this type of behavior are pseudoplastic. At the minimal shear rate, between 0.01 (1/s) and approximately 0.1 (1/s), the diutan gum clearly shows a Newtonian plateau.

**Figure 2:**
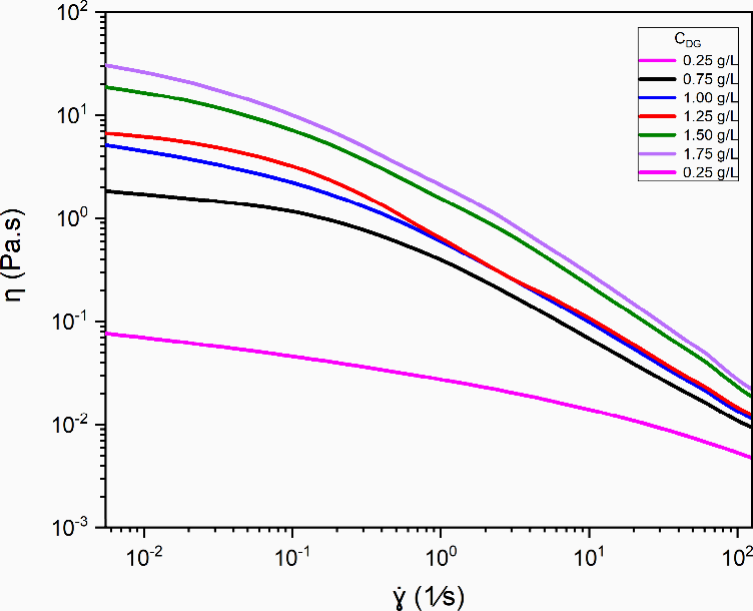
Dependence of the apparent viscosity (η) of diutan in an aqueous solution on the shear rate (γ̇) for different concentrations of *c*_D_ (0, 25, 0.75, 1, 1.25, 1.50, and 1.75 g/L) at 25 °C.

The curves were modeled via a mathematical Carreau–Yasuda fit. Its mathematical form is represented by eq 1. This five-parameter fits very well with a large amount of experimental data, in addition to predicting two constant viscosity plateaus, η_0_, when the shear rate tends to zero, and η_∞_, when the rate tends to infinity.^32^ The term 1/λ_CY_ is a characteristic shear rate that shows that when the fluid departs from the Newtonian plateau η_0_, *a* indicates the transition curvature, and *n* is the power-law index, which measures the shear thinning intensity.

$$\eta =\eta_{\infty }+(\eta_{0}-\eta_{\infty })[1+(\lambda_{\mathrm{CY}}\mathrm{\gamma ̇}{)}^{a}{]}^{n-1/a}$$ 1

Table 1 presents all of the parameters obtained by the model and R-squared values (fit model goodness). The values of *n* < 1 confirm the pseudoplastic behavior of the solutions, which tends to decrease with n further from one as the concentration increases.

**Table 1:** *K* and *n* Indices (Parameters) Obtained from the Carreau–Yasuda Fit for Aqueous Solutions with Diutan Gum at Different Concentrations (0.25, 0.75, 1.00, 1.25, 1.50, and 1.75 g/L) at 25°C

	concentration (g/L)					
CY fits	0.25	0.75	1.00	1.25	1.50	1.75
η_0_	0.07	1.78	4.84	6.57	18.34	29.57
η_∞_	0.005	0.01	0.01	0.01	0.02	0.02
λ_CY_	19.40	5.22	38.31	19.12	46.24	64.20
*a*	0.80	0.86	1.40	1.20	1.51	1.60
*n*	0.60	0.42	0.40	0.28	0.22	0.20
*R* ^2^	0.99	0.99	0.99	0.99	0.99	0.99

Viscoelastic materials can exhibit viscous and elastic behavior in response to force, deformation, and time.^33^ Experiments applied to viscoelasticity include relaxation stress, creep, and oscillatory tests.^34^ From oscillatory tests, the viscoelasticity of polymeric solutions can be inferred from the relationship between the storage modulus (*G*′) and the loss modulus (*G*″). Figure 3 shows the SAOS response of diutan gum solutions in the concentration range from 0.75 to 1.75 g/L. The measurement was not performed at a concentration of 0.25 g/L, as its low viscosity makes it difficult to carry out low-amplitude oscillatory tests in plate–plate and double-gap geometries. The graph with filled squares corresponds to *G*′, and the graph with empty squares corresponds to *G*″, red: 0.75 g/L, blue: 1.00 g/L, purple: 1.25 g/L, green: 1.50 g/L, and black: 1.75 g/L. Each solution has a frequency point where the intersection between the curves (*G*′ and *G*″) occurs. For most concentrations studied, this point occurred at approximately 10 Hz. When the frequency is lower than this value, the *G*′ of the polymer solution is greater than *G*″, indicating that the elastic component dominates the viscoelastic region.^35,36^ At low concentrations, solutions with macromolecules tend to have more viscous behavior because the binding capacity between the polymer’s molecular aggregates is weak, making it difficult for the elastic component to dominate.^37^

**Figure 3:**
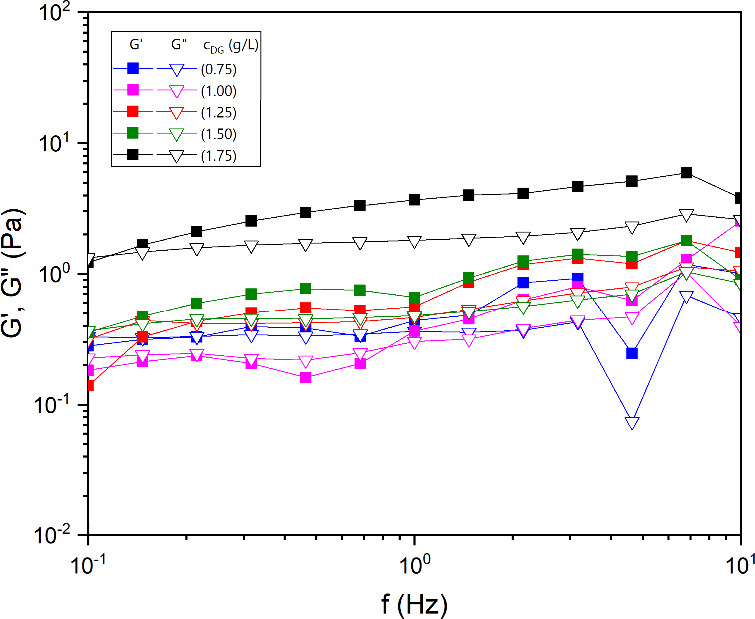
Dependence of the loss modulus (*G*′) and storage modulus (*G*″) for aqueous diutan solutions at different concentrations (0.75, 1, 1.25, 1.50, and 1.75 g/L) at 25 °C.

For all concentrations, *G*′ was greater than *G*″ in most of the frequency range; the elastic component was dominant. This behavior can be attributed to the ability of diutan gum to form a stronger network in aqueous solution through intermolecular associations between the methyl group of L-rhamnose and the oxygen atom of L-rhamnose through van der Waals interactions and hydrogen bonds.^38^ The elasticity of diutan can be attributed to its high molecular weight (5.2 × 10^6^ g/mol) and its ability to retain water within the core of its double helix.^39^

### 3.3 Effects of Salinity

Salt polymer solutions comprise a complex and agglomerated mixture of water molecules, polymer chains, and ions, where each component effectively interacts with the other components.^40^ Generally, when the polymeric solution is in the presence of inorganic salts, cations with opposite charges tend to cover the main chain of the macromolecules. The screening of electrostatic interactions, in turn, allows the chain to bend and assume a smaller and more compact structure, resulting in a decrease in solution viscosity.^41^ In addition, inorganic cations with a substantial degree of hydration help compact the hydrated layer around the polymer molecules, leading to a reduction in viscosity.^42^

Figure 4 shows the relationship between viscosity (η) and shear rate (γ̇) for diutan gum in water at a fixed concentration of 1.75 g/L and for different concentrations of sodium chloride (NaCl) (3, 30, 60, and 90 g/L). All of the samples were subjected to shear thinning, and the viscosity curves practically overlapped at high shear rates, even with increasing salinity. Rheological changes after the addition of a monovalent salt to a polymer solution can be attributed to hydrogen bonds. In the absence of salt, the interactions in the polysaccharide are dominated by electrostatic interactions, which maintain their chains in a relatively extensive configuration. The tendency is for the addition of salt to reduce the viscosity of the solution via the effect of filler screening; at the same time, as the polymer chains approach, there is an increase in the formation of hydrogen bonds between functional groups of the polymer and solvent molecules (such as water). This effect can restrict chain mobility and increase the viscosity of a solution.^43,44^

**Figure 4:**
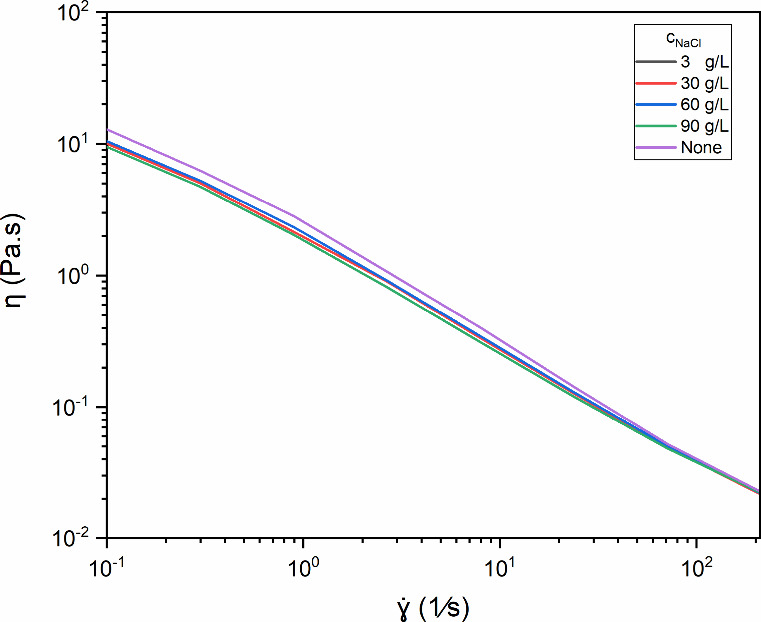
Dependence of the apparent viscosity (η) of diutan in aqueous solution (1.75 g/L) on the shear rate (γ̇) for different NaCl concentrations (3, 30, 60, and 90 g/L) at 25 °C.

In diutan gum, the side chains surround the main chain, forming a helical structure in the presence of water.^45^ The negative charges on the diutan gum are located in the backbone, which is protected by side chains. When the helical conformation is formed, the resulting structure restricts the electrostatic effect.^4^ Nevertheless, the cations (Na^+^) compact the hydrated layer around the molecules; this effect is not very pronounced but can reduce the viscosity of the solution.^20^ The salinity tolerance of diutan gum is attributed mainly to its perfect helical conformation, the location of its side chains, and its ability to retain water in the core of its structure.^3,4,46,30^

Regarding SAOS, Figure 5 shows that the variation in salt concentration (3, 30, 60, and 90 g/L) did not alter the viscoelastic response of diutan gum in solution (with a fixed concentration of 1.75 g/L), with the dominant elastic component (*G*′ > *G*″) in the system. The reduction in the amplitude of the dynamic modulus in the presence of Na^+^ suggests that the diutan gum lost part of its ability to reconstitute the polymeric network after shear, despite maintaining the predominance of the storage modulus (*G*′) over the loss modulus (*G*″).^4^ This behavior did not change with an increasing salt concentration. The results indicate that the solutions are tolerant to salinity, even if the viscoelasticity is partially affected and weak gels are formed. Diutan gum is virtually independent of the ionic environment, whether monovalent or divalent.^4^ In the presence of salt, the molecular chains of diutan gum aggregate to form a robust structure. Although most polysaccharides in brine are covered with inorganic salt crystals, their conformation can still be observed because crystallization occurs only in the lattice structure of the polymer solution.^26,47^

**Figure 5:**
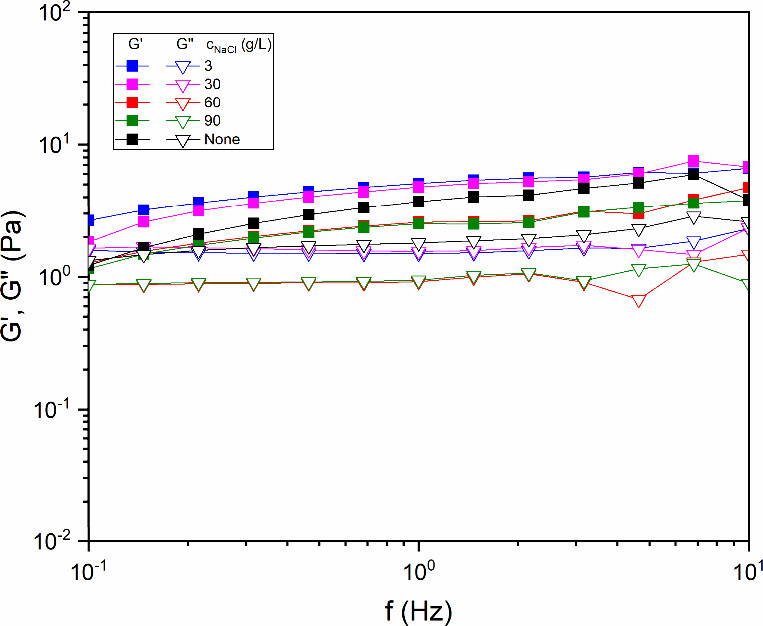
Dependence of the loss modulus (*G*′) and storage modulus (*G*″) for aqueous diutan solutions (1.75 g/L) at different NaCl concentrations (3, 30, 60, and 90 g/L) at 25 °C.

The results presented in this section are derived from accelerated aging tests conducted under SA conditions and DA agitation (600 rpm), providing complementary insights into the thermal and mechanical stability of diutan gum for drilling fluid applications. Figure 6 shows the relationship between viscosity (η) and shear rate (γ̇) for diutan gum in water at a fixed concentration of 1.75 g/L with different concentrations of sodium chloride (NaCl) (140,000 and 180,000 mg/L). The “None” label refers to the control sample without added salt and aged (600 rpm).

**Figure 6:**
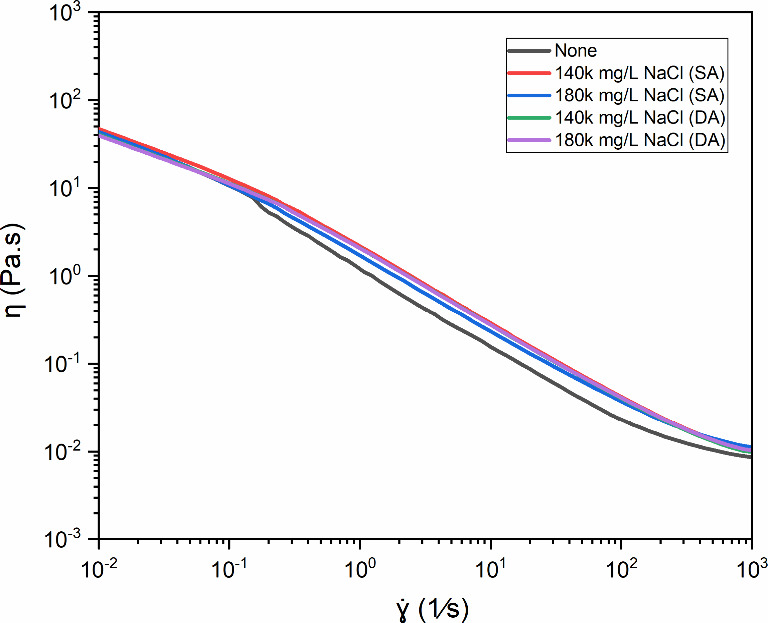
Shear rate (γ̇) dependence of apparent viscosity (η) for diutan gum (1.75 g/L) at various NaCl concentrations (none, 140,000, and 180,000 mg/L) after 16 h of static (SA) and dynamic aging (DA) at 25 °C (Adapted from Santos, *DGMK Conference Proceedings* (2025), pp. 294–305, with permission).

The flow behavior remained unchanged despite agitation and high salinity (up to 180,000 mg/L NaCl), with nearly overlapping viscosity curves. This remarkable salt tolerance stems from the stable helical structure, optimal side chain distribution, and exceptional water retention capacity of the diutan gum within its molecular framework. Figure 7 shows the SAOS response of diutan gum solutions under different salinity conditions and aging methods. In the graph, the filled squares represent *G*′ (storage modulus), whereas the open squares correspond to *G*″ (loss modulus). The color code indicates the following: green (None), light blue (140k mg/L static aging), dark blue (140k mg/L DA), red (180k mg/L static aging), and purple (180k mg/L DA). Each solution has a characteristic crossover frequency where *G*′ and *G*″ intersect. For most concentrations studied, this crossover occurred at approximately 10 Hz. Below this frequency, the polymer solution’s *G*′ exceeds *G*″, demonstrating elastic-dominated viscoelastic behavior.

**Figure 7:**
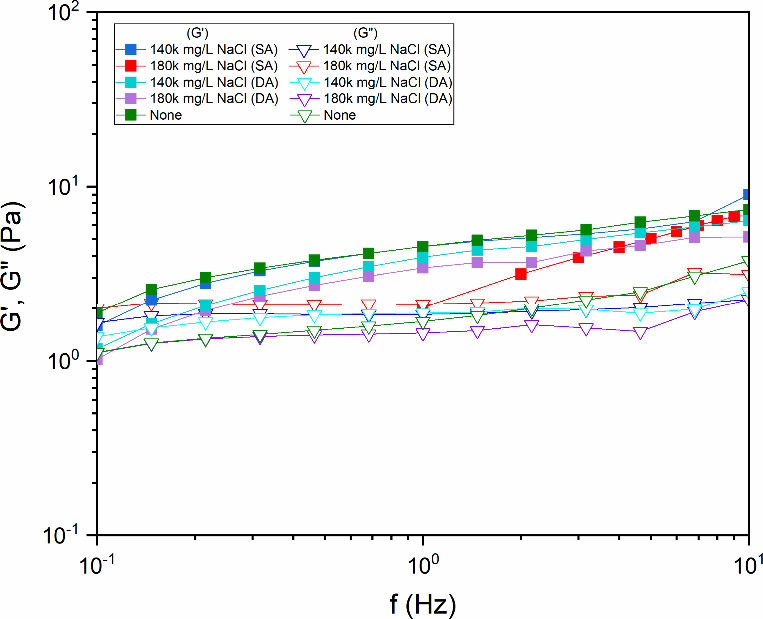
Frequency dependence of the loss modulus (*G*″) and storage modulus (*G*′) for aqueous diutan gum solutions at different NaCl concentrations (140k and 180k mg/L) subjected to static (SA) and dynamic (DA) aging tests for 16 h at 25 °C. “None” refers to the control sample without temperature variation or aging treatment (Adapted from Santos, *DGMK Conference Proceedings* (2025), pp. 294–305, with permission).

The incorporation of NaCl demonstrates a negligible effect on the elastic characteristics of diutan gum relative to its viscous behavior at frequencies of less than 10 Hz. This phenomenon occurs because salt ions do not substantially interfere with the hydrophilic interactions between side chains and water molecules within the helical structure. Remarkably, even under high shear conditions (up to 600 rpm), the viscoelastic properties of diutan gum remain unaffected by the addition of salt. This exceptional stability arises from the helix’s robust architecture, which resists shear-induced degradation while maintaining its water-entrapping functionality.^4,7,10,11^ The ionic strength introduced from NaCl fails to disrupt the critical hydrogen-bonded network within the helical core, thereby preserving elastic dominance regardless of the applied mechanical stress. Consequently, the structural framework maintains its integrity, sustaining an elastic predominance at low frequencies and under shear conditions.

### 3.4 Effect of Temperature

Thermal stability is related to the chemical structure of the polymer chain, and its mobility determines essential characteristics of the product, such as its hardness or brittleness. This mobility is a function of the agitation of the atoms in the molecules and is directly proportional to the temperature. Figure 8 shows the flow curves of aqueous solutions containing a fixed concentration of 1.75 g/L diutan gum at temperatures of 25, 40, 60, and 80 °C. The solutions are subjected to shear thinning at all working temperatures. The tendency is that with increasing temperature, the viscosity of aqueous solutions decreases because of van der Waals forces and hydrogen bonds, which help form macromolecular tangles and are weakened due to the increase in the movement of the molecules.^48^

**Figure 8:**
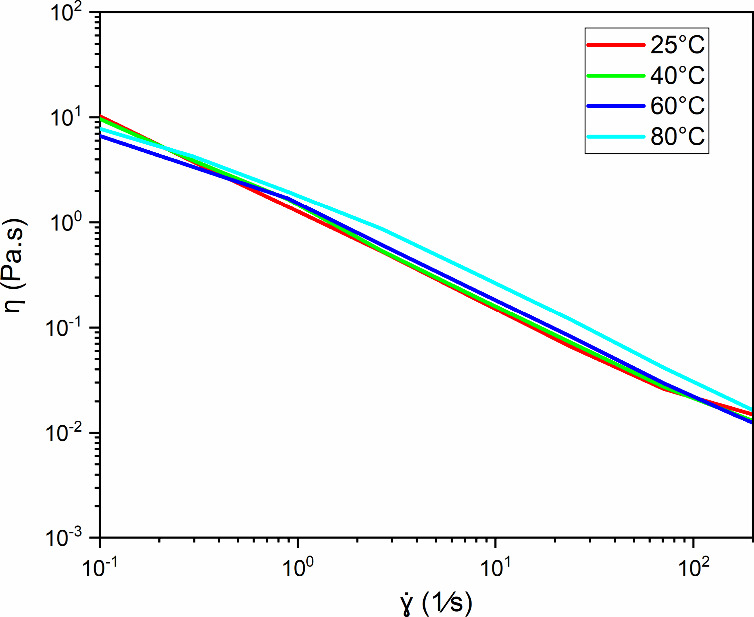
Dependence of the apparent viscosity (η) of diutan gum in aqueous solution (1.75 g/L) on the shear rate (γ̇) at different temperatures (25, 40, 60, and 80 °C).

Reference 15 reported an increase in the viscosity of aqueous solutions of diutan gum, even with increasing temperature. They noted that the flow rate of diutan gum is independent of temperature and that its flow rate is non-Newtonian, even when the temperature varies from 10 to 60 °C. Compared with other gums, such as xanthan gum and konjac gum, diutan gum is thermally more stable, and this stability can be attributed to its double helix conformation and its high ability to retain water within its nucleus.^20^ Figure 9 shows the viscoelastic response of solutions containing diutan gum at different temperatures. The storage modulus (*G*′) was greater than the loss modulus (*G*″) for all temperatures, indicating that the elastic component is dominant in the flow in the frequency range from 0.1 to 10 Hz. The amplitude between the modules and the effect of NaCl indicated the formation of well-organized but weak network structures.

**Figure 9:**
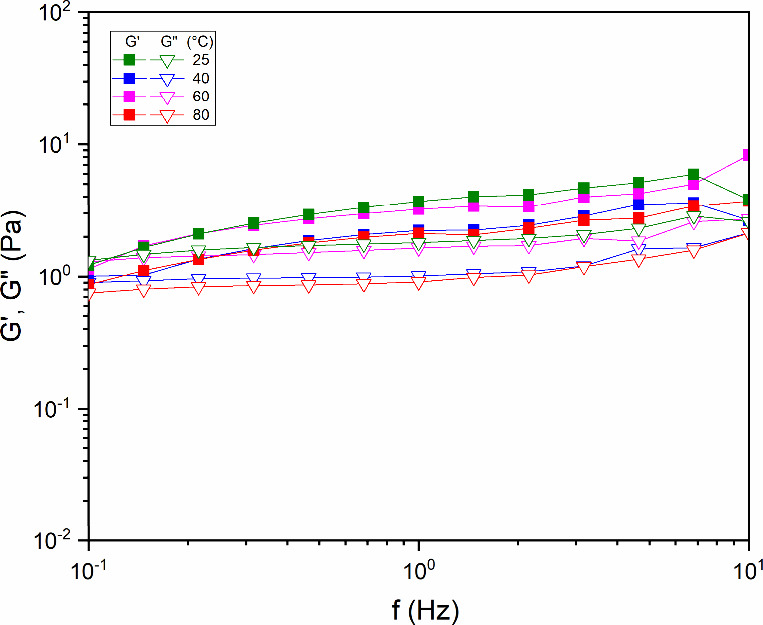
Dependence of the loss modulus (*G*′) and storage modulus (*G*″) for aqueous diutan solutions (1.75 g/L) at different temperatures (25, 40, 60, and 80 °C).

The modules *G*′ and *G*″ were not sensitive to thermal gain, a factor that can be attributed to the double helix conformational structure of the diutan gum. Typically, at high temperatures, the water molecules adhering to the periphery of the helices detach and escape, because of the weakening of the hydrogen bonds and the thermal movement of the molecules. However, the double helix of diutan gum maintains its conformation because it has a rigid molecular structure capable of draping most water molecules inside its nucleus, preventing their escape and maintaining constant water retention.^49^

The results presented in this section are derived from accelerated aging tests conducted under static conditions and dynamic agitation (600 rpm), providing complementary insights into the thermal and mechanical stability of diutan gum for drilling fluid applications. Figure 10 presents the flow curves obtained for aqueous solutions containing a fixed concentration of 1.75 g/L diutan gum at 60 and 140 °C subjected to SA and DA. The pseudoplastic behavior was maintained under all of the evaluated conditions, except for DA at 600 rpm and 140 °C, which exhibited Newtonian fluid behavior. A significant viscosity reduction occurred under these conditions, indicating polymer chain degradation.

**Figure 10:**
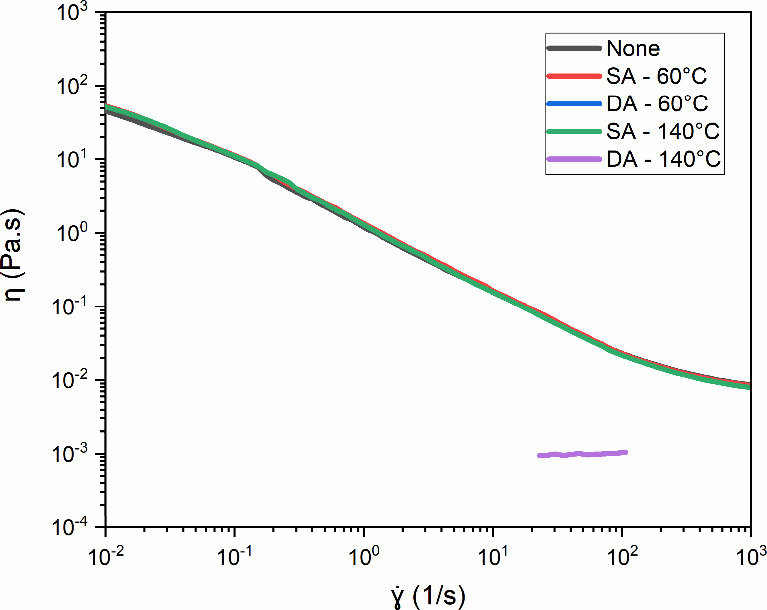
Apparent viscosity (η) of diutan gum in aqueous solution (1.75 g/L) as a function of shear rate (γ̇) at different temperatures (60 and 140 °C) after static (SA) and dynamic (DA) aging tests for 16 h at 25 °C. “None” refers to the control sample without temperature variation or aging treatment (Adapted from Santos, *DGMK Conference Proceedings* (2025), pp. 294–305, with permission).

Figure 11 presents the SAOS behavior of the solutions. Across the entire temperature range studied, the storage modulus (*G*′) consistently remained higher than the loss modulus (*G*″), demonstrating that the elastic component of the material predominated over the viscous component. This behavior was observed within the tested frequency range of 0.1 to 10 Hz, further confirming the solid-like, elastic-dominated rheological response of the system.

**Figure 11:**
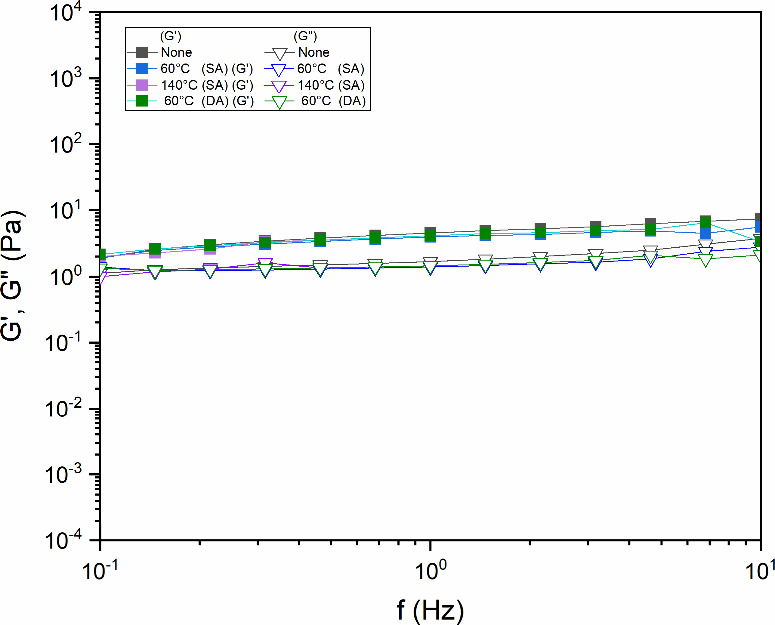
Viscoelastic moduli (*G*′, *G*″) versus frequency for diutan gum solutions (1.75 g/L) after thermal aging at 60 and 140 °C under both static (SA) conditions and dynamic (DA) agitation (600 rpm) for 16 h. The unaged control (“None”) was maintained at a constant temperature without aging (Adapted from Santos, *DGMK Conference Proceedings* (2025), pp. 294–305, with permission).

The *G*′ and *G*″ moduli showed no sensitivity to a thermal increase for samples heated at 60 °C with SA and DA or at 140 °C with static aging. This behavior can be attributed to the double-helical conformational structure of the diutan gum. Typically, water molecules adsorbed on the helical periphery at high temperatures detach and escape due to weakened hydrogen bonds and increased molecular thermal motion. However, the double helix of DNA maintains its conformation because of its rigid molecular structure, which traps most water molecules within its core, preventing their release and maintaining constant hydration.^15^ For the solution heated to 140 °C with DA (600 rpm), *G*′ and *G*″ were not measured, as the sample’s behavior approached that of a Newtonian fluid, making it difficult to obtain accurate measurements in a plate–plate and double gap geometry. This behavior suggests a transition from a viscoelastic to a pure viscous fluid state, likely caused by thermal degradation effects promoting hydrogen bond rupture. Furthermore, magnetic stirring induces shear stresses that can break bonds and disrupt molecular aggregates.^50^

## 4 FTIR of Aqueous Solutions

Diutan gum presents a band centered at 3420 cm^–1^ attributed to the stretching vibration of the O–H groups, and the peaks at 2934 and 2885 cm^–1^ correspond to the vibrations of the asymmetric and symmetric stretching of the carbon and hydrogen bonds, respectively.^31,51,51^ At approximately 1730 cm^–1^, a significant band was attributed to the absorption of C=O bonds in the acetyl groups of diutan gum. The peaks at 1234 and 1026 cm^–1^ are attributed to the tensile vibrations of C–O–C and C–O–H. In addition, the peaks at 1610 and 1365 cm^–1^ are attributed to the asymmetric and symmetrical vibrations of the COO group.^11^ Figure 12 shows the effect of increasing the concentration of NaCl in the solution on the chemical structure of the diutan gum.

**Figure 12:**
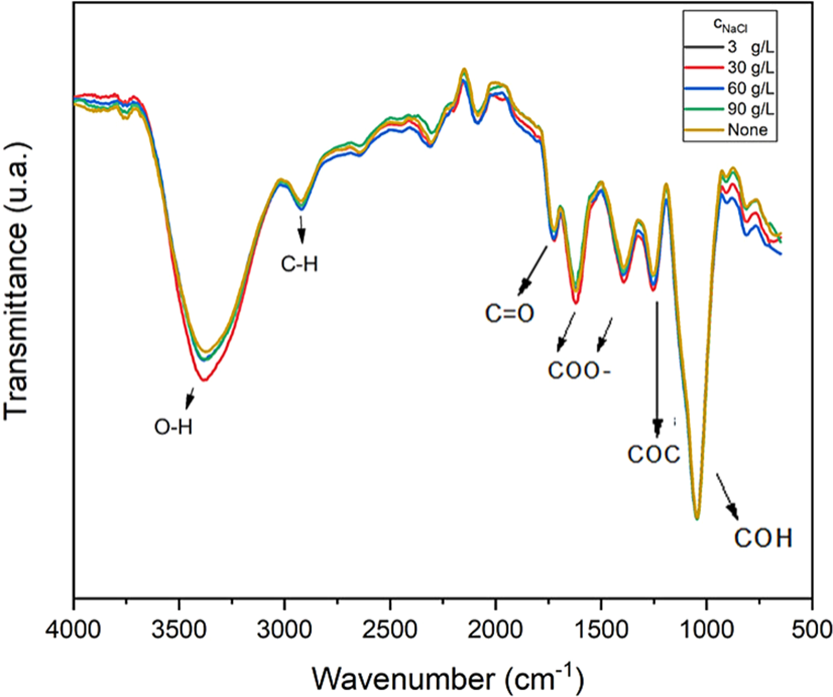
FTIR spectra of aqueous diutangum solutions (1.75 g/L) at different NaCl concentrations (3, 30, 60, and 90 g/L) at 25 °C.

For all concentrations, the bands corresponding to the respective functional groups (O–H, C–H, C=O, COC, COH, and COO^–^) present in the polymer chain were maintained, and the same was true for the spectrum corresponding to the temperature variation, as illustrated in Figure 13.

**Figure 13:**
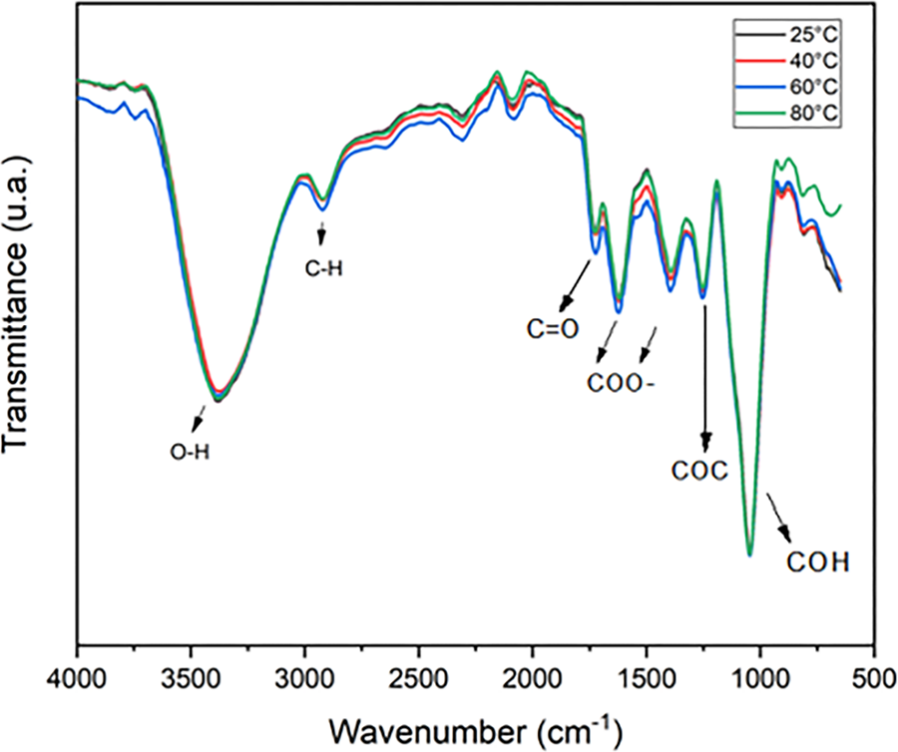
FTIR spectra of aqueous diutan solutions (1.75 g/L) at different temperatures (25, 40, 60, and 80 °C).

This maintenance of the polymer chain and its functional groups, even with variations in the salinity and temperature of the medium, indicates that the rheological behavior found, especially that of viscosity, is directly related to the organization and alignment of the polymers with the flow without losses occurring. Functional groups or some structural impairment of the chain. Aqueous solutions containing diutan gum are stable to changes in salinity and temperature, a behavior mainly attributed to its double helix conformation, location of side chains, and water holding capacity.^4,11,15,16,52,53^

Figure 14 presents the FTIR spectra of diutan gum under the following conditions: (DIUTAN GUM_GD) unaged, (GD_EE_140 °C) after SA at 140 °C, and (GD_ED_140 °C) after DA at 140 °C and 600 rpm. The analysis aims to verify possible changes in the polymeric structure due to DA, since the flow curve (Figure 10) indicated a loss of pseudoplastic behavior and a transition to a Newtonian profile under these conditions.

**Figure 14:**
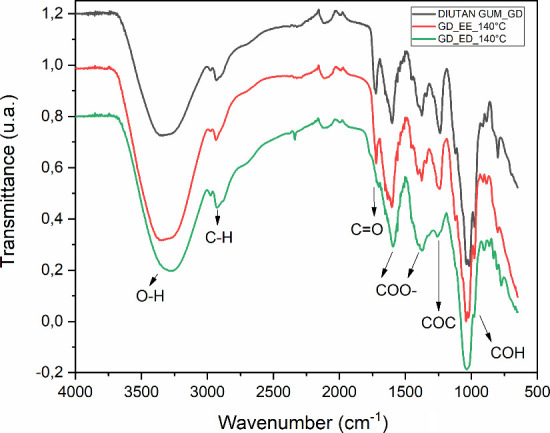
FTIR spectra of aqueous diutan gum solutions (1.75 g/L) under different temperature conditions (unaged, static, and dynamic aging).

As presented in Figure 14, the ED_140 sample exhibits a noticeable attenuation of the absorption band associated with the carboxylate C=O stretching vibration at 1720 cm^–1^, along with a reduction in intensity at 1250 cm^–1^, attributed to C–O–C stretching vibrations characteristic of glycosidic bonds. Concurrently, there was an increase in the intensity of the bands corresponding to the deprotonated carboxylate groups (COO^–^), which were observed as a doublet near 1400 and 1600 cm^–1^. These spectral changes can be interpreted as indicative of chemical modifications associated with hydrolysis and oxidation mechanisms, which promote the degradation of the polymer backbone and a consequent reduction in molar mass. These changes in the FTIR pattern have been reported for other polysaccharides, such as xanthan gum, starch, and cellulose.^54–56^

## 5 Conclusions

TGA revealed that diutan gum shows two distinct stages of thermal events: initial water desorption at approximately 100 °C and polymer decomposition between 230–274 °C, corresponding to 40% mass loss. The high decomposition onset at 230 °C exceeds typical thermal requirements for drilling fluids (<200 °C), demonstrating outstanding thermal stability suitable for high-temperature, high-pressure (HTHP) applications. The rheological results revealed that the aqueous solutions of diutan gum exhibited pseudoplastic behavior under all the conditions analyzed, confirming its suitability as a polymeric additive for aqueous drilling fluids. It was also stable for different salinities and temperatures, indicating that it applies to deep drilling through geothermal systems and Brazilian presalt wells. The predominance of the storage modulus (*G*′) over the loss modulus (*G*″) in the frequency range of 0.1–10 Hz indicates the ability of diutan to form organized structures, even at low concentrations (0.75 g/L). However, the low amplitude between these modules suggests the formation of weak gels, which are liable to rupture under high shear rates and may be advantageous for applications in drilling fluids that require adaptive rheological control.

Under static conditions (SAs), the rheological properties (apparent viscosity and viscoelastic moduli *G*′ and *G*″) of diutan gum are maintained even at extreme salinities (180,000 mg/L NaCl) and high temperatures (140 °C). In contrast, dynamic aging (DA) (600 rpm) at 140 °C caused significant rheological degradation, as confirmed by the FTIR results. The combined effects of thermal stress and mechanical shear progressively disrupted hydrogen bonds and, to a lesser extent, secondary covalent interactions that stabilized the helical structure. This synergistic mechanism led to network breakdown, viscosity reduction, and loss of elasticity. The absence of degradation under static conditions at the same temperature highlights the critical role of mechanical shear in the degradation process. Spectroscopic analyses via FTIR revealed the structural stability of diutan gum without changes in its functional groups, demonstrating that the salinity and temperature were insufficient to degrade its chemical structure. This stability is attributed to the helical conformation of the diutan gum, which protects the polymer chain and allows the retention of water inside it, contributing to the maintenance of the rheological properties of the system.

Thus, the results show that diutan gum has a high resistance to adverse temperature and salinity conditions, which are fundamental characteristics for its application as an additive in drilling fluids. Its ability to maintain favorable rheological properties, biodegradability, and environmentally friendly characteristics reinforces its potential as a sustainable alternative for the petroleum industry. Future studies should explore in more detail the impact of other operational parameters on its performance, with the aim of optimizing its application under field conditions.

## ⊥

Institute of Drilling Engineering and Fluid Mining (IBF), TU Bergakademie Freiberg (TUBAF), 09599 Freiberg, Germany
